# Fluconazole Disk Diffusion on CHROMagar Candida Plus for Differentiation Between *Candidozyma auris* and *Candida parapsilosis*

**DOI:** 10.3390/jof12080606

**Published:** 2026-08-13

**Authors:** Sarah Israel, Jonathan Lellouche, Loujin Ardda, Ariel Israel, Sharon Amit, Ronen Ben-Ami, Itzhack Polacheck, Jacob Moran-Gilad, Maya Korem

**Affiliations:** 1 Faculty of Medicine, Hebrew University of Jerusalem, Jerusalem 9101002, Israel; sarahi@hadassah.org.il (S.I.); lojainriadarda@gmail.com (L.A.); itzhackp@ekmd.huji.ac.il (I.P.); giladko@post.bgu.ac.il (J.M.-G.); 2 Department of Clinical Microbiology and Infectious Diseases, Hadassah Medical Center, Jerusalem 9101002, Israel; 3 Clinical Laboratories Department, Sanz Medical Center, Netanya 4244916, Israel; jlellouche@laniado.org.il; 4 Adelson School of Medicine, Ariel University, Ariel 4070000, Israel; 5 Leumit Research Institute, Leumit Health Services, Tel Aviv 6473817, Israel; aisrael@leumit.co.il; 6 Department of Epidemiology and Preventive Medicine, School of Public Health, Gray Faculty of Medical & Health Sciences, Tel Aviv University, Tel Aviv 6997801, Israel; 7 Clinical Microbiology Laboratory, The Chaim Sheba Medical Center, Ramat Gan 5266202, Israel; sharon.amit@sheba.health.gov.il; 8 Gray Faculty of Medicine and Health Sciences, Tel Aviv University, Tel Aviv 6997801, Israel; ronenba@tlvmc.gov.il; 9 Infectious Diseases Unit, Tel Aviv Sourasky Medical Center, Tel Aviv 6997801, Israel

**Keywords:** *Candidozyma auris*, *Candida parapsilosis*, CHROMagar Candida Plus, fluconazole disk diffusion, phenotypic identification, antifungal susceptibility testing

## Abstract

Background: This study evaluated the utility of fluconazole disk diffusion on CHROMagar Candida Plus for differentiating *Candidozyma auris* from *Candida parapsilosis*. Methods: In this in vitro study, clinical isolates of both *C. auris* and *C. parapsilosis* were used. Antifungal susceptibility testing was performed via broth microdilution and disk diffusion with different fluconazole concentrations on both CHROMagar Candida Plus and RPMI agar. Colony color was also assessed. Results: This study demonstrates high categorical agreement between disk diffusion on CHROMagar Candida Plus and broth microdilution for both species. An optimal cut-off zone diameter of 10 mm was identified on CHROMagar Candida Plus with 50 µg fluconazole, providing 91% sensitivity and 90% specificity for differentiating the two species. Notably, at 24 h, 83 out of 88 (94.3%) *C. auris* isolates exhibited a bluish, blue-green, or turquoise coloration on the reverse side of the plate, suggesting that reverse-side colony color on CHROMagar may serve as a supportive phenotypic indicator when combined with disk diffusion testing, to differentiate *C. auris* from *C. parapsilosis* in the laboratory setting. Conclusions: Fluconazole disk diffusion on CHROMagar Candida Plus, supplemented by reverse-side colony coloration, offers a practical, low-cost adjunctive strategy to aid in the differentiation of *C. auris* and *C. parapsilosis* in clinical laboratories.

## 1. Introduction

*Candidozyma auris* (*C. auris*) is a multidrug-resistant yeast associated with healthcare outbreaks, high mortality in bloodstream infections, and persistent environmental contamination. Its resistance to commonly used antifungals and ability to evade standard identification methods make it a major challenge for infection control and clinical management [1,2]. The CDC recommends screening high risk patients, typically by swabbing the groin and axilla to detect *C. auris* colonization [3]. Accurate and timely identification during screening is important, as it directly informs isolation measures and antifungal stewardship strategies [4].

CHROMagar™ Candida Plus is commonly used for presumptive identification of *C. auris* based on its distinctive colony color [5]. However, other studies highlighted its limitations, particularly in differentiating *Candida parapsilosis* (*C. parapsilosis*) from *C. auris*, with 44% of *C. parapsilosis* isolates erroneously reported as *C. auris* [6,7]. Accurate differentiation between these two species is critical for both clinical management and infection control. Clinically, *C. parapsilosis* remains largely susceptible to fluconazole, whereas *C. auris* exhibits high rates of fluconazole resistance, making rapid differentiation vital for guiding appropriate empiric antifungal therapy [8]. From an infection control perspective, misidentifying *C. parapsilosis* as *C. auris* leads to unnecessary, resource-intensive patient isolation, contact tracing, and PPE expenditure, whereas failing to recognize *C. auris* risks uncontained nosocomial outbreaks [8].

While supplementation of CHROMagar Candida Plus with fluconazole has been shown to improve species discrimination in former studies [6,7], these approaches did not assess inhibition zone diameters around a fluconazole disk, which may provide additional phenotypic information while simultaneously generating susceptibility data.

To address this diagnostic gap, we evaluated whether incorporating a fluconazole disk onto CHROMagar Candida Plus plates could enhance the medium’s discriminatory power. By leveraging the characteristic reduced fluconazole susceptibility often observed in *C. auris* relative to other *Candida* species, we aimed to develop a simple, cost-effective method to improve species-level identification. This approach may be particularly valuable in low resource settings that lack access to MALDI-TOF and sequence-based diagnostics, while also offering complementary susceptibility-related information in well-resourced laboratories.

For clarity, consistency with the existing literature, and ease of reading, the species currently classified as *Candidozyma auris* is referred to throughout this manuscript as *C. auris*.

## 2. Materials and Methods

### 2.1. Study Setting and Ethics

This study was conducted at the Department of Clinical Microbiology and Infectious Diseases, Hadassah Hebrew-University Medical Center, Jerusalem, Israel. The study was approved by the Hadassah Medical Organization Institutional Review Board (Hadassah IRB), Jerusalem, Israel (Approval No. HMO-0460-12; approved on 22 September 2020).

### 2.2. Fungal Isolates

We analyzed patient-unique *C. parapsilosis* blood isolates (*n* = 42) and *C. auris* clinical isolates (*n* = 89, axilla/groin surveillance swabs [*n* = 75], urine and sputum cultures [*n* = 14]), collected between 2014 and 2024 from four hospitals in Israel. Clade classification was available for 47 of the 89 *C. auris* isolates (Clade I, *n* = 4; Clade II, *n* = 4; Clade III, *n* = 34; Clade IV, *n* = 5), having been previously determined by MLST at the national central mycology reference laboratory, according to a previously described procedure [9].

All isolates were stored at −80 °C until testing. Prior to testing, isolates were thawed and cultured on Sabouraud dextrose agar (SDA; HyLabs, Rehovot, Israel) for 24–48 h. Species-level identification was confirmed by sequencing the internal transcribed spacer (ITS1-5.8S-ITS2) region [10,11], and comparing the obtained sequences with reference sequences in MycoBank (https://www.mycobank.org), and by using the AurisID qPCR Kit (OLM Diagnostics, Newcastle upon Tyne, UK) [12].

Quality control strains were *C. parapsilosis* ATCC 22019 and *C. auris* ATCC MYA-5001 (Manassas, VA, USA).

### 2.3. Antifungal Susceptibility Testing (AST)

All isolates underwent fluconazole susceptibility testing using broth microdilution (reference method) and disk diffusion on agar media [CHROMagar Candida Plus, and RPMI 1640 (HyLabs, Rehovot, Israel)].

### 2.4. Broth Microdilution Testing

Broth microdilution (BMD) was performed according to Clinical and Laboratory Standards Institute (CLSI) guidelines. Fluconazole concentrations ranged up to 256 µg/mL, exceeding the CLSI upper limit of 64 µg/mL [13]. Results for *C. parapsilosis* were interpreted using CLSI minimal inhibitory concentration (MIC) breakpoints [14]: susceptible ≤ 2 µg/mL, susceptible dose-dependent 4 µg/mL and resistant ≥ 8 µg/mL. For *C. auris*, no established clinical breakpoints exist to date. Based on available data indicating high MICs (>16 µg/mL) [15] a tentative CLSI breakpoint of ≥32 µg/mL was used to define resistance [16].

### 2.5. Disk Diffusion Testing

Inoculum preparation and disk diffusion testing followed CLSI M44-Ed3 methodology [17]. Briefly, colonies grown on SDA were suspended in sterile water and adjusted to a cell density of 0.5 McFarland standard (~10^6^ CFU/mL) [17].

Suspensions were then inoculated onto CHROMagar Candida Plus (Hy-labs, Rehovot, Israel) and, in parallel, onto RPMI 1640 agar supplemented with MOPS and 2% Glucose [18], using sterile cotton swabs to ensure even distribution. RPMI agar was used as a comparator medium, as it is routinely used for antifungal susceptibility testing in our laboratory and has been shown to produce zone diameters that correlate with BMD results as well as those obtained using Mueller–Hinton agar [19].

For disk diffusion testing, two fluconazole disk potencies were evaluated [17]:

(1) Commercial 25 μg disks (Pro-Lab Diagnostics, Richmond Hill, ON, Canada).
(2) In-house 50 μg disks prepared by applying 25 μL of a 2 mg/mL fluconazole solution onto 6 mm blank disks (Pro-Lab Diagnostics, Richmond Hill, ON, Canada), followed by drying prior to use [17].

Disks were placed at the center of each inoculated plate. Disk performance was verified using quality control strains and reproducibility across replicates.

Plates were incubated at 35 °C up to 48 h. Inhibition zone diameters were measured in millimeters (mm) using a calibrated ruler after 24 h and interpreted according to CLSI criteria [14]. All zone measurements were performed with the investigators blinded to the species identity of the isolates.

For *C. parapsilosis*, zones diameters of ≥17 mm were classified as susceptible, 14–16 mm as susceptible-dose dependent (S-DD), and ≤13 mm as resistant [14]. For *C. auris* isolates, a zone diameter of ≤11 mm was considered resistant [20].

Inoculated plates without fluconazole disks served as growth controls.

### 2.6. Colony Color Assessment

Colony color on CHROMagar Candida Plus was recorded after 24 and 48 h of incubation. According to the manufacturer, *C. auris* typically produces light blue colonies with a surrounding blue halo on both sides of the plate. No standardized color description is available for *C. parapsilosis* [21,22].

### 2.7. Repeatability and Error Definitions

All tests in this study were performed in duplicate. In cases of discrepancy, the experiment was repeated and performed in triplicate.

Discrepancies between disk diffusion and broth microdilution results were categorized as follows: (1) Very major error (VME): disk diffusion susceptible, BMD resistant (2) Major error (ME): disk diffusion resistant, BMD susceptible (3) Minor error (mE): disk diffusion S-DD, while BMD is either susceptible (S) or resistant (R). Rates of VMEs, MEs, and mEs were calculated as follows: VME rate: number of VMEs divided by the number of resistant isolates × 100; ME rate: number of MEs divided by the number of susceptible isolates × 100; mE rate: number of mEs divided by the total number of isolates tested **×** 100. For analysis purposes, susceptibility was defined as both susceptible and susceptible dose-dependent.

### 2.8. Statistical Methods

Agreement between broth microdilution (BMD), MIC values and disk diffusion zone diameters for fluconazole was assessed using a combination of graphical and quantitative methods. Scatter plots were generated to visualize the relationship between BMD MIC values (*x*-axis) and disk diffusion zone diameters (*y*-axis). Separate plots were created for each combination of fungal species (*C. auris* and *C. parapsilosis*), fluconazole disk concentration (25 µg and 50 µg), and growth medium (CHROMagar Candida Plus and RPMI agar). To evaluate the ability of disk diffusion zone diameters to differentiate *C. auris* from *C. parapsilosis*, receiver operating characteristic (ROC) curves were constructed. These analyses were performed for each combination of growth medium (CHROMagar Candida Plus and RPMI agar) and fluconazole disk concentration (25 µg and 50 µg). Zone diameter was designated as the diagnostic variable, with *C. auris* defined as the positive class. For each condition, the Area Under the Curve (AUC), sensitivity, specificity, and the optimal zone diameter cutoff value were determined.

All statistical analyses and visualizations were performed using python 3.11. A *p*-value < 0.05 was considered statistically significant.

## 3. Results

### 3.1. Distribution and Susceptibility of Fungal Isolates

Of the 42 *C. parapsilosis* isolates, 25 (59.5%) were susceptible to fluconazole, 2 (4.8%) were susceptible dose-dependent, and 15 (35.7%) were resistant. Among the 89 *C. auris* isolates, 79 (88.8%) were resistant to fluconazole, while 10 (11.2%) were susceptible.

**Figure 1:**
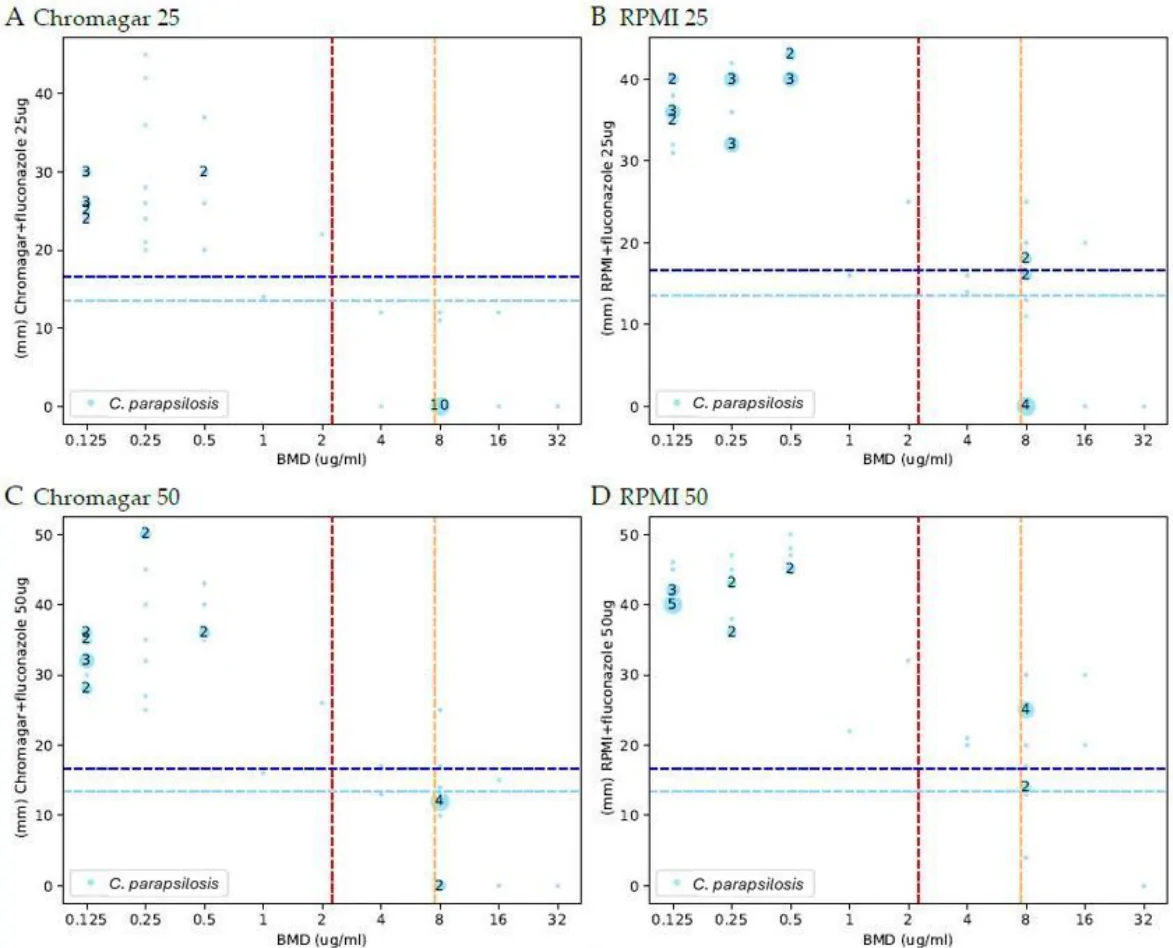
Agreement between fluconazole broth microdilution MICs and disk diffusion zone diameters for *Candida parapsilosis* isolates. Scatter plots display fluconazole MIC values (*x*-axis) versus corresponding disk diffusion zone diameters (*y*-axis) for *C. parapsilosis* isolates. Each subplot represents a distinct combination of growth medium (CHROMagar Candida Plus or RPMI) and fluconazole disk concentration (25 µg or 50 µg). Vertical dashed lines indicate CLSI-defined BMD susceptibility breakpoints; horizontal dashed lines represent corresponding disk diffusion interpretive breakpoints. Points are colored by interpretative category: red/dark blue = Susceptible, orange/light blue = Susceptible-Dose Dependent. Numbers denote overlapping isolates.

**Figure 2:**
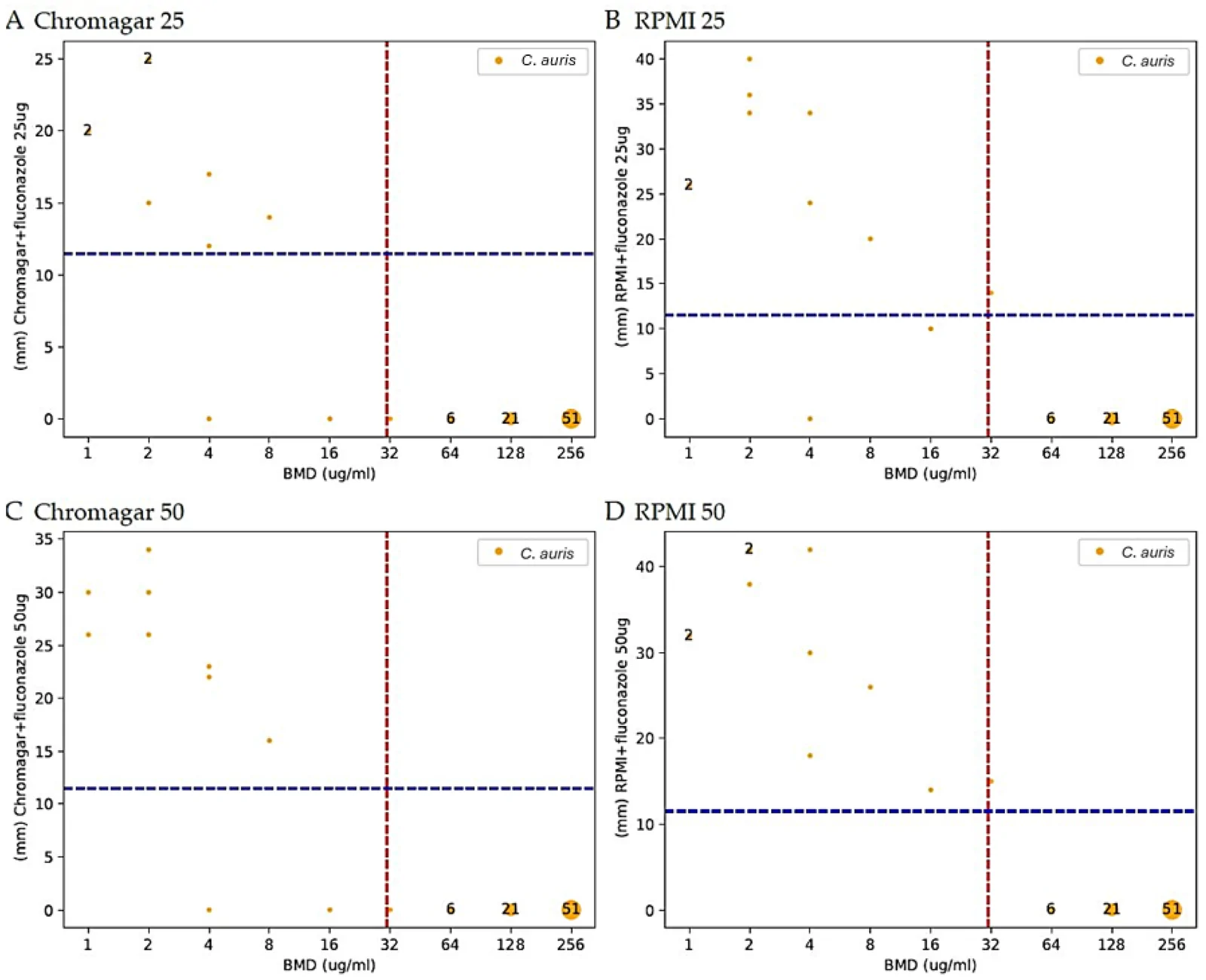
Agreement between fluconazole broth microdilution MICs and disk diffusion zone diameters for *Candidozyma auris* isolates. Scatter plots comparing fluconazole MIC values (*x*-axis) with disk diffusion zone diameters (*y*-axis) for *C. auris* isolates across four testing conditions: CHROMagar Candida Plus or RPMI medium, each with fluconazole at 25 µg or 50 µg. CLSI tentative resistance breakpoints (MIC ≥ 32 µg/mL; zone diameter ≤ 11 mm) are indicated by vertical and horizontal dashed lines. Overlapping data points are labeled with the number of isolates sharing identical values.

For *C. parapsilosis*, CHROMagar Candida Plus with 25 µg fluconazole performed best, showing a categorical agreement of 95.2% with BMD, with two MEs and no VMEs. In contrast, CHROMagar Candida Plus with 50 µg fluconazole, as well as RPMI with 25 µg and 50 µg, demonstrated lower categorical agreement and yielded 2, 5, and 10 VMEs, respectively. For *C. auris*, both 25 µg and 50 µg fluconazole disks on CHROMagar Candida Plus achieved high categorical agreement with BMD (97.7%). Two MEs were noted at each concentration, but no VMEs occurred. RPMI with 25 µg and 50 µg showed similar categorical agreement, however, both yielded one VME, and RPMI 25 µg additionally produced two MEs. The susceptibility testing results and associated error rates for all tested conditions are summarized in Table 1 and Figure 1 and Figure 2.

Among Clade I isolates, three of four (75%) were resistant to fluconazole. One isolate showed a ME, being categorized as susceptible by BMD but resistant by disk diffusion on CHROMagar plates with 25 or 50 µg fluconazole and on RPMI with 25 µg fluconazole. All four Clade II isolates were susceptible to fluconazole. In Clade III, 32 of 34 isolates (94.1%) were resistant, whereas in Clade IV, three of five isolates (60%) were resistant. No errors were observed across susceptibility testing methods for Clades II, III, and IV.

### 3.2. Differentiating C. parapsilosis and C. auris

**Figure 3:**
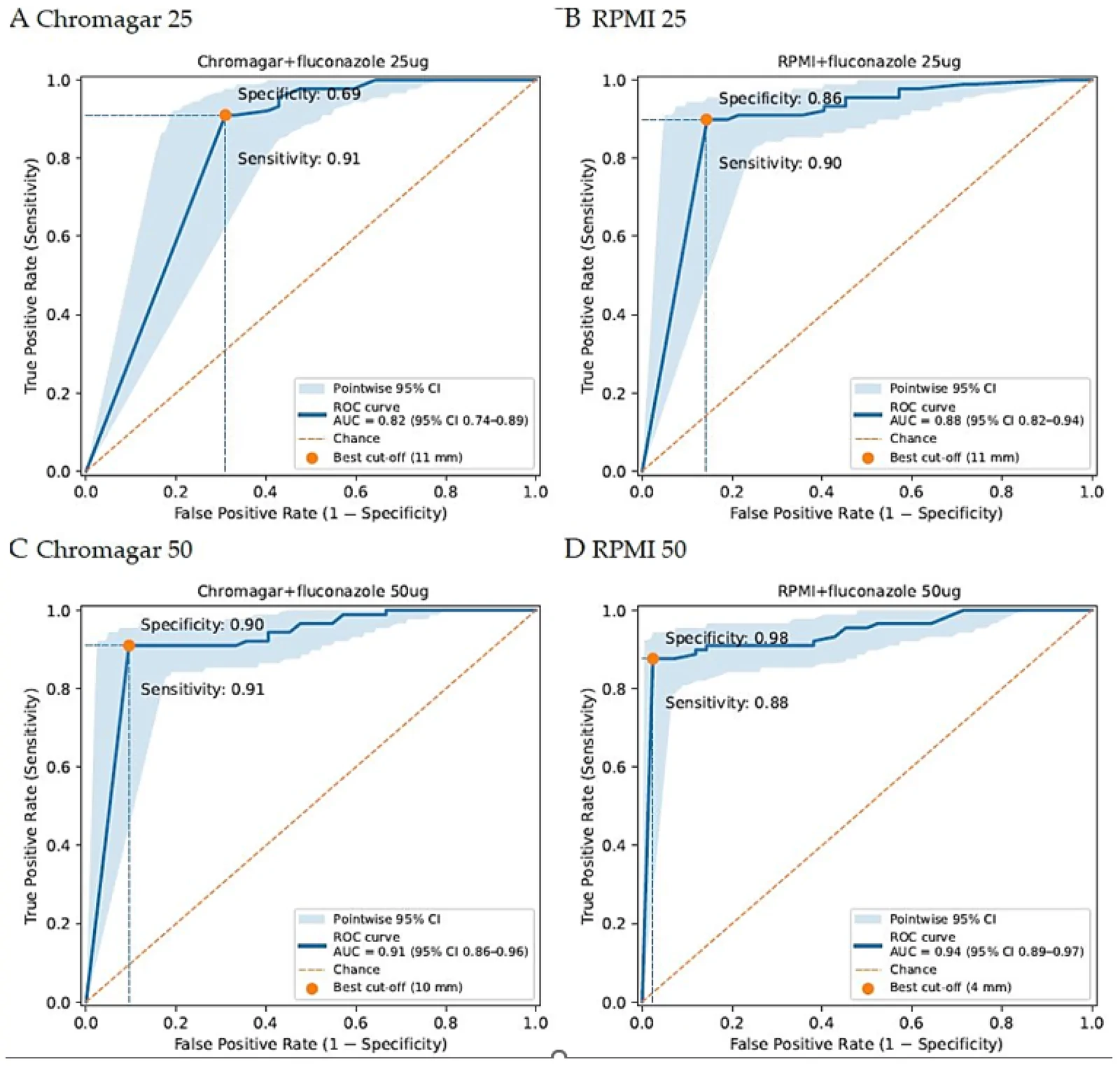
Receiver Operating Characteristic (ROC) curve analysis for differentiating *Candidozyma auris* from *Candida parapsilosis* using fluconazole disk diffusion zone diameters. Receiver operating characteristic (ROC) curves evaluating the diagnostic performance of fluconazole disk diffusion zone diameters in distinguishing *C. auris* (positive class) from *C. parapsilosis* across four conditions: CHROMagar Candida Plus and RPMI media, each with 25 µg and 50 µg fluconazole disks. Area under the curve (AUC), optimal cutoff zone diameter, sensitivity, and specificity are shown for each agar and fluconazole disk combination.

ROC curves for differentiating *C. auris* from *C. parapsilosis* based on fluconazole disk diffusion zone diameters are presented in Figure 3. AUC values ranged from 0.82 to 0.94 across the four tested conditions (CHROMagar Candida Plus and RPMI media, each with either 25 µg or 50 µg fluconazole disks), indicating good diagnostic performance for species discrimination. Optimal zone diameter cut-offs varied between 4 mm and 11 mm, depending on the medium and fluconazole concentration. The use of a higher fluconazole concentration (50 µg) generally improved both specificity and AUC, particularly on RPMI agar.

For differentiating *C. auris* (positive class in the ROC analysis) from *C. parapsilosis*, CHROMagar Candida Plus with 50 µg fluconazole yielded the best performance with an optimal cutoff of 10 mm providing 91% sensitivity and 90% specificity (Figure 3C). Using this method, 8 of 89 *C. auris* isolates (9%) would be missed (false negatives), and 4 of 42 *C. parapsilosis* isolates (9.52%) would be incorrectly classified as *C. auris* (false positives).

### 3.3. Colony Color Differentiation on CHROMagar Candida Plus

Colony color assessment on CHROMagar Candida Plus was available for 88 *C. auris* and 35 *C. parapsilosis* isolates after 24 and 48 h of incubation at 35 °C. Color characteristics were not systematically recorded for every isolate during the early phase of evaluation, as these observations were initially part of routine workflow. Consequently, analysis of colony color was restricted to the subset of isolates with retrievable retrospective color data.

**Figure 4:**
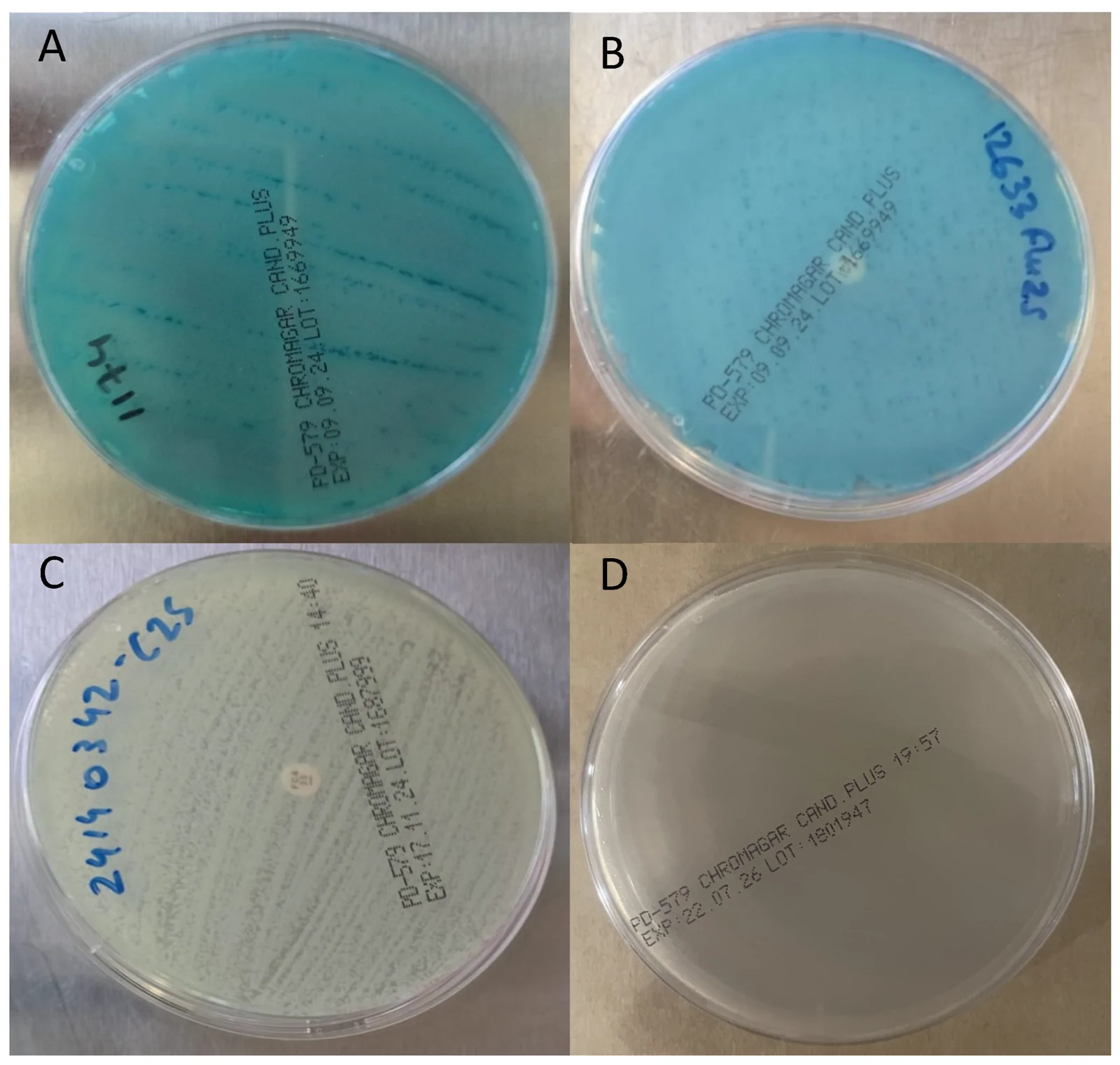
Colonies of isolates on the reverse side of CHROMagar Candida Plus plates after 24 h of incubation: *Candidozyma auris* exhibiting turquoise or bluish coloration (**A**,**B**), *Candida parapsilosis* exhibiting white coloration (**C**), and control CHROMagar Candida Plus plate (**D**).

At 24 h, 83/88 (94.3%) *C. auris* isolates exhibited bluish, blue-green, or turquoise coloration on the colony reverse, whereas none of the *C. parapsilosis* isolates showed this pattern (0/35; *p* < 0.0001, Fisher’s exact test) (Figure 4). In contrast, surface coloration at 24 h was cream-colored in all *C. parapsilosis* isolates and in 37/88 *C. auris* isolates, limiting its discriminatory value.

By 48 h, bluish, blue-green, or turquoise coloration on the colony reverse was observed in all 88 *C. auris* isolates and in 28/35 *C. parapsilosis* isolates. Surface coloration at this time was cream in 63/88 and cream-pink in 25/88 *C. auris* isolates, whereas 33/35 *C. parapsilosis* isolates displayed a cream-pink appearance. Overall, colony color characteristics at 48 h were not useful for distinguishing between the two species.

Reverse-side bluish, blue-green, or turquoise coloration at 24 h demonstrated excellent discriminatory performance, with a sensitivity of 94.3% and specificity of 100% for the identification of *C. auris*. This performance was comparable to that of CHROMagar Candida Plus supplemented with 50 µg fluconazole using a 10 mm inhibition zone cutoff, which achieved a sensitivity of 91% and specificity of 90%.

The two phenotypic markers provided complementary information. Of the five *C. auris* isolates lacking characteristic reverse coloration at 24 h, four were correctly identified by complete inhibition of fluconazole disk diffusion (0 mm zone diameter), whereas one isolate exhibited a 22 mm inhibition zone and remained misclassified by both methods. Conversely, although all *C. parapsilosis* isolates lacked the characteristic reverse coloration, four isolates showed unexpectedly small inhibition zones (three with 0 mm zones and one with a 10 mm zone diameter) and were therefore falsely classified as *C. auris* by the disk diffusion criterion.

Combining the two markers using a positive result defined by either characteristic reverse-side coloration or a fluconazole inhibition zone diameter < 10 mm increased sensitivity to 98.9% (87/88), at the expense of a reduction in specificity to 88.6% (31/35).

## 4. Discussion

This study demonstrates the potential of fluconazole disk diffusion on CHROMagar Candida Plus as a practical alternative for laboratories without access to MALDI-TOF, enabling reliable differentiation between *C. parapsilosis* and *C. auris*. In addition to species identification, this approach provides antifungal susceptibility data with reduced turnaround time. The high categorical agreement observed between disk diffusion and BMD for both species indicates that when performed according to CLSI guidelines and interpreted with species-specific criteria, disk-diffusion can yield reliable results. Notably, the absence of VMEs on CHROMagar Candida Plus with the 25 µg fluconazole disk is clinically significant, as it reduces the risk of underestimating resistance and misguiding treatment.

The ROC curve analysis further supports the discriminatory value of this method. AUC values above 0.8 indicate that fluconazole disk diffusion zone diameters provide meaningful separation between *C. auris* and *C. parapsilosis*. Using a 50 µg fluconazole disk on CHROMagar Candida Plus, a 10 mm cutoff yielded 91% sensitivity and 90% specificity for distinguishing the two species. Although these metrics are encouraging, the presence of 9% false negatives (missed *C. auris*, potentially delaying infection control measures) and 10% false positives (misclassified *C. parapsilosis*, possibly leading to unnecessary isolation precautions), underscores the need for cautious interpretation. Importantly, the observed misclassification rate of approximately 9–10% is clinically relevant in the context of *C. auris* surveillance, where even small proportions of false-negative results may delay infection control interventions, and false-positive results may lead to unnecessary isolation measures and resource utilization. Therefore, while the method shows strong overall performance, it should be considered a rapid screening tool rather than a definitive standalone diagnostic method.

The observation of distinct color differences adds a valuable clue for presumptive identification. Specifically, the turquoise/blue-green coloration observed on the reverse of *C. auris* colonies after 24 h, absent in *C. parapsilosis* under the same conditions, could enhance discriminatory accuracy when combined with fluconazole disk diffusion results. When combined using a positive rule defined as either characteristic reverse-side coloration or a fluconazole inhibition zone diameter < 10 mm, the overall performance improved sensitivity to 98.9% (87/88) but reduced specificity to 88.6% (31/35). These findings suggest that reverse-side colony coloration is best used as a highly specific first-line marker, while fluconazole disk diffusion may serve as a complementary screening tool to improve detection of atypical *C. auris* isolates. Such complementary phenotypic clues may be especially valuable in laboratories with limited access to MALDI-TOF or molecular diagnostics, reinforcing the role of this approach as a practical screening tool.

This study has limitations. Although the isolate collection was diverse, it may not fully capture the global genetic variability of *C. auris* and *C. parapsilosis*, particularly given the well-documented differences in antifungal resistance among *C. auris* clades. Fluconazole susceptibility varies substantially across clades: Clade I (South Asia) and Clade III (South Africa, now prevalent in Europe and North America) are frequently resistant, making the discriminatory ability of CHROMagar Candida Plus combined with fluconazole disk diffusion potentially valuable. In regions where fluconazole-susceptible *C. auris* Clade II is prevalent (e.g., East Asia), both *C. auris* and *C. parapsilosis* may exhibit larger inhibition zone diameters, potentially reducing the reliability of the established cutoff. Clade IV (South America) and V (Iran) also display variable susceptibility patterns [23], underscoring the need for a broader validation across geographically diverse collections. From a local perspective, a recent national outbreak in Israel was predominantly driven by a clade III clone, which was significantly more resistant to fluconazole than non-Clade III isolates [24]. This suggests that the 10 mm cutoff would likely be effective in the Israeli context for differentiating *C. auris* from *C. parapsilosis*. Future studies should explore the incorporation of additional antifungal disks or alternative chromogenic media formulations to enhance discrimination in regions with a high prevalence of fluconazole-susceptible *C. auris* clades. An additional limitation relates to the increasing global prevalence of fluconazole-resistant *C. parapsilosis*, which may impact the generalizability of the proposed cutoff values. Rising resistance rates in certain healthcare settings could lead to reduced inhibition zone diameters in *C. parapsilosis*, thereby decreasing the specificity of fluconazole-based discrimination between the two species. This evolving resistance landscape underscores the need for periodic recalibration of interpretive breakpoints and local validation before implementation in routine diagnostic workflows. Nevertheless, approximately 35% of the *C. parapsilosis* isolates included in this study were fluconazole resistant, suggesting that the proposed cutoff retained discriminatory performance despite a relatively high prevalence of resistance in the comparator group. Another limitation of this study is the unequal number of isolates evaluated, with a larger number of *C. auris* compared with *C. parapsilosis* isolates. Although ROC analysis is not affected by disease prevalence, the smaller comparator group may reduce the precision of performance estimates. Validation in larger, independently collected and more balanced isolate collections is warranted.

From a clinical perspective, fluconazole disk diffusion on CHROMagar Candida Plus represents a cost-effective and accessible method for differentiating of *C. auris* from *C. parapsilosis*. Interpretable results can be available in 24–48 h, supporting timely infection control interventions. The complementary observation of reverse-side colony coloration further enhances ease of use and strengthens the discriminatory value of this approach in laboratories lacking advanced identification technologies.

## 5. Conclusions

This study shows that fluconazole disk diffusion on CHROMagar Candida Plus is a practical tool for differentiating *C. auris* from *C. parapsilosis*. The method demonstrated strong agreement with broth microdilution, particularly for *C. auris* (97.7% at 25 µg) and *C. parapsilosis* (95.2% at 25 µg) on CHROMagar Candida Plus. A 10 mm cutoff with a 50 µg fluconazole disk achieved 91% sensitivity and 90% specificity for identifying *C. auris*, while the consistent turquoise reverse-side colony coloration observed for *C. auris* provides an additional marker. Together, these features support rapid presumptive identification within 24–48 h, aiding timely infection control and treatment decisions, especially in resource-limited settings.

## Figures and Tables

**Table 1:** Evaluation of fluconazole disk diffusion methods against CLSI broth microdilution for *C. parapsilosis* and *C. auris* isolates.

	BMD	Agar, Fluconazole Disk Concentration	Susceptibility ^a^	Resistance	Categorical Agreement	Very Major Error	Major Error	Minor Error
*C. parapsilosis* (*n* = 42)	S: *n* = 25 (59.5%) S-DD: *n* = 2 (4.8%) R: *n* = 15 (35.7%)	CHROMagar Candida Plus, 25 µg	25 (59.5%)	17 (40.5%)	40 (95.2%)	0	2 (7.4%)	0
		CHROMagar Candida Plus, 50 µg	30 (71.4%)	12 (28.6%)	37 (88.1%)	2 (13.3%)	1 (3.7%)	2 (4.8%)
		RPMI, 25 µg	34 (81%)	8 (19%)	35 (83.3%)	5 (33.3%)	0	2 (4.8%)
		RPMI, 50 µg	39 (92.9%)	3 (7.1%)	30 (71.4%)	10 (66.6%)	0	2 (4.8%)
*C. auris* (*n* = 89)	S: *n* = 10 (11.2%) R: *n* = 79 (88.8%)	CHROMagar Candida Plus, 25 µg	8 (9.0%)	81 (91%)	87 (97.7%)	0	2 (20%)	
		CHROMagar Candida Plus, 50 µg	8 (9.0%)	81 (91%)	87 (97.7%)	0	2 (20%)	
		RPMI, 25 µg	9 (10.1%)	80 (89.9%)	86 (96.6%)	1 (1.3%)	2 (20%)	
		RPMI, 50 µg	11 (12.4%)	78 (87.6%)	88 (98.9%)	1 (1.3%)	0	

^a^ Susceptibility was defined as both susceptible (S) and susceptible dose-dependent (S-DD). CLSI-defined BMD susceptibility breakpoints for *C*. *parapsilosis*: Susceptible (S), MIC ≤ 2 µg/mL; susceptible dose-dependent (S-DD), MIC = 4 µg/mL; resistant (R), MIC ≥ 8 µg/mL; Disk diffusion interpretive breakpoint for *C. parapsilosis*: S, ≥17 mm; S-DD, 14–16 mm; R, ≤13 mm. CDC tentative BMD resistance breakpoint for *C. auris*: Resistant (R), MIC ≥ 32 µg/mL. Disk diffusion interpretive breakpoint: Resistant (R), zone diameter ≤ 11 mm.

## Data Availability

The original contributions presented in this study are included in the article. Further inquiries can be directed to the corresponding author.
